# Principle of Least Psychomotor Action: Modelling Situated Entropy in Optimization of Psychomotor Work Involving Human, Cyborg and Robot Workers

**DOI:** 10.3390/e20110836

**Published:** 2018-10-31

**Authors:** Stephen Fox, Adrian Kotelba

**Affiliations:** VTT Technical Research Centre of Finland, FI-02044 VTT, Finland

**Keywords:** artificial intelligence, autonomous, craft, cyborg, future of work, human, industrial, manual work, psychomotor, robot, skills, sustainability, work, worker

## Abstract

Entropy in workplaces is situated amidst workers and their work. In this paper, findings are reported from a study encompassing psychomotor work by three types of workers: human, cyborg and robot; together with three aspects of psychomotor work: setting, composition and uncertainty. The Principle of Least Psychomotor Action (PLPA) is introduced and modelled in terms of situated entropy. PLPA is founded upon the Principle of Least Action. Situated entropy modelling of PLPA is informed by theoretical studies concerned with connections between information theory and thermodynamics. Four contributions are provided in this paper. First, the situated entropy of PLPA is modelled in terms of positioning, performing and perfecting psychomotor skills. Second, with regard to workers, PLPA is related to the state-of-the-art in human, cyborg and robot psychomotor skills. Third, with regard to work, situated entropy is related to engineering of work settings, work composition and work uncertainty. Fourth, PLPA and modelling situated entropy are related to debate about the future of work. Overall, modelling situated entropy is introduced as a means of objectively modelling relative potential of humans, cyborgs, and robots to carry out work with least action. This can introduce greater objectivity into debates about the future of work.

## 1. Introduction

The word autonomous was first used more than 200 years ago. In the 21st Century, autonomous is often used to refer to industrial and service robots that are intended to carry out psychomotor work without direct human control [1,2]. However, autonomous is also used to describe human psychomotor work skills [3]. In particular, when humans have mastery of psychomotor work skills, they can perform them autonomously. They can carry out psychomotor work successfully with little, if any, conscious thought. This is very different to novices who have to have supervision and have to apply much conscious thought when performing psychomotor work [4]. Another important characteristic of autonomous human psychomotor work skills is that physical motions are carried out with the irreducible simplicity of fluid elegance [5,6]. Furthermore, when humans have developed autonomous psychomotor work skills, they have adaptive expertise that enables them to transfer their skills to new situations. This is very different to novices whose physical motions are more clumsy than elegant, and are limited to one situation such as training class [7,8,9,10]. Autonomous human psychomotor skills are an example of embodied cognition where the physical body has a constitutive role in cognitive processing. For example, a tall craftsperson may come to adopt automatically a slightly different starting position to a shorter craftsperson in order to perform a task action elegantly. Thus, autonomous psychomotor skills are not a set of standard actions, but rather individual embodied repertoires that share characteristics with the embodied repertoires of others [11,12,13].

Psychomotor skills range from fine to gross. Fine skills involve very precise neuromuscular coordination, such as when threading a needle. Gross skills can involve whole body movement, for example, when swinging a sledgehammer [14]. Overall, human autonomous psychomotor work skills are characterized by least action. This is because little, if any, conscious thought is required and physical motions are characterized by the irreducible simplicity of fluid elegance [4,5,6,15]. This is in stark contrast to the carrying out of psychomotor work by robots, which can involve intensive energy consumption for both computational and mechanical effort [16]. Nonetheless, autonomous human psychomotor work skills are often disregarded in debates about the future of work. Rather, a common theme of debate is the order in which human psychomotor skills will be replaced by robots that perform psychomotor skills [17,18,19].

However, studies indicate that there is much work where robots workers add most value when they are employed together with human workers [20,21]. Moreover, the potential of robots to take over all psychomotor work is limited by the huge diversity of needs for psychomotor skills around the world [22,23]. Accordingly, a unifying principle is introduced in this paper for autonomous psychomotor work skills; in particular, a unifying principle that encompasses human workers, cyborg workers, and robot workers. Here, cyborgs are humans who are enhanced by permanent implanting and/or persistent wearing of work technologies [24]. Together, the combination of human workers, cyborg workers and robot workers can introduce hybrid intelligence systems that deploy natural and artificial intelligence [25].

The remainder of the paper comprises six sections. In Section 2, the unifying principle is introduced. This is founded upon the Principle of Least Action [26,27]. It is stated as the Principle of Least Psychomotor Action (PLPA) and is expressed in terms of situated entropy. In Section 3, PLPA expressed in terms of situated entropy is related to the state-of-the-art in human, cyborg and robot psychomotor work skills. In Section 4, progress towards PLPA through engineering of work settings, work composition, and work uncertainty is modelled in terms of situated entropy. In Section 5, examples are provided of PLPA modelling. In Section 6, implications from modelling PLPA are discussed from the perspectives of theory building, applied research, and practice. In Section 7, conclusions are stated. The overall contribution of this paper is to introduce situated entropy as means of objectively modelling the relative potential of humans, cyborgs, and robots to carry out work with the least amount of action. This can contribute introducing greater objectivity into debates about the future of work.

## 2. Principle of Least Psychomotor Action

### 2.1. Problem Statement

Ideally there would be a unifying principle to provide an objective basis for modelling the relative potential of humans, cyborgs and robots to carry out work. Thus far, however, the reality is that decision making about whether to invest in humans, cyborgs or robots is not based on any objective principle for modelling. As a consequence, investment decisions are not based on objective consideration of the relative merits of different types of workers, and expensive capital investment failures follow. For example, investments in robotics at Tesla slowed down production and its costly investment in robotics was removed from its factory [28]. More broadly, debate about the future of work is driven by hype about “the rise of the robots”: rather than objective consideration of the relative merits of different types of workers [17,18,19]. Hence, it is proposed that a unifying principle for modelling is introduced, which is founded upon the objective scientific basis provided by the Principle of Least Action. This widely applied principle is founded upon scientific observations that nature tends to act as simply as possible: for example, by taking a path between two points that requires the least action. Indeed, it has been argued that it is “Nature’s Command” to “follow the path of least action” [29]. The Principle of Least Action has been found to be able to simplify explanation of a wide range of highly complex phenomena involving physical motion [26,30,31]. Moreover, The Principle of Least Action can be used in optimization problems involving human intervention [27].

### 2.2. PLPA

**Figure 1:**
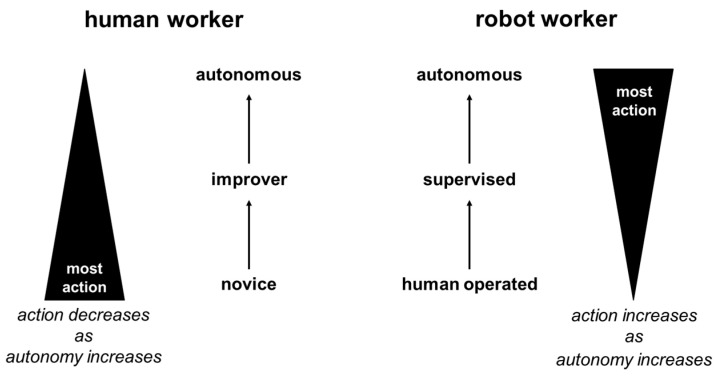
Contrasting levels of action in human and robot psychomotor work in 2018.

As summarized in Figure 1, in contrast with robot autonomous psychomotor work skills, least action is a fundamental attribute of human autonomous psychomotor work skills. Although embodied cognition eschews separation of mind and body [11,12,13], from the point-of-view of an observer of psychomotor work there is what can be seen (i.e., what is external) and what cannot be seen (i.e., what is internal) [32]. Externally, physical motions are characterized by the irreducible simplicity of fluid elegance. Also, the need for external human supervision decreases as human novices progress to being human experts who can work autonomously. Internally, little, if any, conscious thought is required in familiar settings. Also, little, if any, additional thought is required when transferring psychomotor skills to new settings. [3,7,9,10]. Furthermore, there is evidence that advanced human psychomotor skill repertoires improve judgement about skill related objects; support prediction of the actions of skill related others; and increase ability to comprehend action-related language [33]. Overall, it can be argued that autonomous human psychomotor skills involve external independence and internal automacity. As can be seen by an observer of psychomotor work, the skilled human works independently of external supervision in carrying out tasks, while internally, the skilled human works with automacity enabled by psychomotor memory [34]. By contrast, while the need for external human supervision decreases as robots become more autonomous, the amount of computation increases as robots become more autonomous. For example, there are heavy computational loads involved in transferring what has been learnt in one setting, such as a university robotics laboratory, to diverse work settings [16].

**Figure 2:**
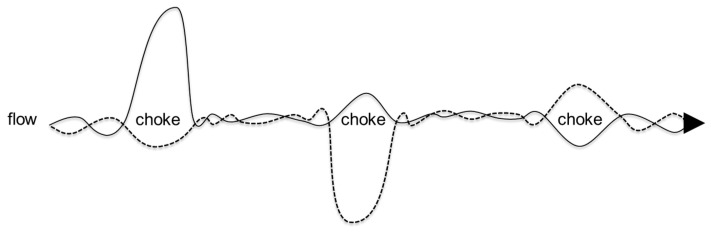
Least psychomotor action in the flow state of autonomous action.

Whether work is undertaken by human, cyborg or robot workers, carrying out work with the least amount of action can improve ecological sustainability through lowest energy consumption and wear and tear, etc. Moreover, social sustainability issues in the division of labor can be addressed if there are comparative analyses of the relative potential of human, cyborg and robot workers to carry out work with the lowest amount of action. Hence, it is appropriate for the Principle of Least Action to provide the basis for a unifying principle for psychomotor skills, which encompasses both the internal (i.e., the “psycho” in psychomotor skills) and the external (i.e., the “motor” in psychomotor skills). As illustrated in Figure 2, internal action and external action can be merged together when humans are in the “flow” of autonomous action. When this “flow” is interrupted by conscious thought, autonomous action is “choked” and then there is separation between the internal action and the external action of psychomotor work [35,36]. This is summarized in the words of psychologist George Humphrey as follows: No man skilled at a trade needs to put his constant attention on the routine work. If he does, the job is apt to be spoiled [37].

In Figure 2, internal action is indicated by the dotted line, while external action is illustrated by the solid line. In the first choke state, external action is high but productivity is low: for example, when a worker tries out several different positions before undertaking performance of a psychomotor skill. In the second choke state, internal action is high but productive is low: for example, when a worker engages in conscious thought while undertaking performance of a psychomotor skill. In the third choke state, both internal action and external action increase but productivity is low: for example, when a worker repeats a motion in mind and in body while trying to perfect a psychomotor skill. In the flow state, by contrast, productivity is high while internal action and external action are low.

Accordingly, the principle of least psychomotor action (PLPA) can be stated as follows: the preferred combination of workers is the combination of worker types that can carry out psychomotor work with the least internal action and least external action. When related to human workers, internal action includes conscious thought, if any, in familiar settings and in the transfer of skills to new settings. When related to robot workers, internal actions include computational effort in familiar setting and in the transfer of skills to new settings. For human workers, external action includes biomechanical motion. For robot workers, external action includes mechatronic motion. If supervision is required, as it is for human novice workers and robot worker that supervised by humans, it is appropriate to take into account the internal and external actions of supervision.

Total psychomotor action *S* can be described as the sum of external (mechanical) action *S_e_* and the internal (information processing) action *S_i_* as follows:

*S* = *S_e_* + *S_i_* (1)

*S_e_* is described in Equation (2) where *KE*(*t*) denotes kinetic energy and *PE*(*t*) denotes the potential energy of an actuator, such as a human hand or robot arm

(2) $$\;S_{e}=\int_{t_{1}}^{t_{2}}\left[KE\left(t\right)-PE\left(t\right)\right]dt\;$$

*S_i_* is described in Equation (3) where *E*(*t*) denotes the energy used for information-processing tasks. Information-processing tasks could include, for example, perception, recognition, and computation.

(3) $$\;S_{i}=\int_{t_{1}}^{t_{2}}E\left(t\right)dt\;$$

Here, situated entropy modelling of PLPA is informed by theoretical studies concerned with connections between information theory, thermodynamics, and circuit design. Namely, we modify the principle of least computational action proposed in [38]. More specifically, we observe that spatial entropy of [38] is equivalent to situated entropy in workplaces. Furthermore, we assume that the energy used for information processing tasks *E* is proportional to the square of the information complexity of those tasks, which are measured by situated entropy *H* [39]. Furthermore, we assume that worker may receive additional side information *I* that needs to be processed, for example, additional instructions from human supervisor or from information embedded in a component by an electronic tag or similar. Consequently,

(4) $$\;E\left(t\right)=c_{H}\left(t\right){\left[H\left(t\right)\right]}^{2}+c_{I}\left(t\right){\left[I\left(t\right)\right]}^{2}\;$$

where *c_H_*(*t*) and *c_I_*(*t*) denote, respectively, the energy cost of processing one bit of information related to complexity of the task and the energy cost of processing one bit of information related to side information. We make the distinction between *c_H_*(*t*) and *c_I_*(*t*) because in some scenarios, processing of side information could be very energy efficient or incur no cost at all. Thus, internal action *S_i_* can be rewritten as

(5) $$\;S_{i}=\int_{t_{1}}^{t_{2}}\left\{c_{H}\left(t\right){\left[H\left(t\right)\right]}^{2}+c_{I}\left(t\right){\left[I\left(t\right)\right]}^{2}\right\}dt\;$$

Psychomotor work skills encompass positioning, performing and perfecting. In doing so, human psychomotor work skills draw extensively upon innate human psychomotor abilities. For example, when getting into position to perform psychomotor work, people deploy their spatial perception and physical balance. Also, reflection for perfecting of future psychomotor performance draws upon innate human capabilities evolved to enable adaptation of psychomotor performance for new environmental conditions. Reflection about refinement and expansion of psychomotor skills is very different to conscious attention to routine movement in familiar settings, which interrupts and impairs autonomous performance of psychomotor skills [31,32,33,34]. The performance of psychomotor work skills can often be likely to depend more upon work skill-specific training than does getting into position and reflection upon how to perfect [40,41,42]. In order to facilitate comprehensive balanced assessment of PLPA, positioning, performance and perfecting aspects of psychomotor work should be included.

For example, let us assume that completion of a given task requires three steps: positioning *X*, performing the task *Y*, and perfecting the procedure or outcome *Z*. In general, there will always be differences between workers, time instants, and spatial locations reflecting different ways in which these steps are executed. For that reason, we model those steps as random variables. The total situated entropy *H* can be represented as joint entropy of random variables modeling positioning, performing, and perfecting steps, or, by chain law of entropy

(6) H=H(X, Y, Z)=H(X)+H(Y|X)+H(Z|Y,X) 

We model the complexity of performing a given task using a conditional entropy *H*(*Y*|*X*). Modelling of positioning *H*(*X*) and perfecting H(Z|Y,X) aspects of psychomotor work is more complex. Friston’s information-based model [43,44] is drawn upon to describe quantitatively positioning and perfecting aspects of psychomotor work. Friston’s information-based model offers a description of a wide range of cognitive processes such as perception, learning or inference and is particularly accurate in explaining perceptual processes [45]. Fristons’s model is applied in more detail in Section 4 of this paper. 

The total action *S* = *S_e_* + *S_i_* is minimized by jointly optimizing mechanical motions described by *S_e_*, cost of computations modeled by *c_H_*(*t*) and *c_I_*(*t*), and situated entropy modeled by *H*(*X*,*Y*,*Z*). Note that the cost of processing *c_H_*(*t*) and *c_I_*(*t*) may depend on who or what is performing the information processing tasks, for example, human, cyborg, robot. The situated entropy, on the other hand, mainly depends on the engineering work settings, work composition, and work uncertainty [46].

## 3. Workers: PLPA and Human, Cyborg, Robot Psychomotor Skills

Equations (1)–(6) can be applied in modelling any type of worker. The current state-of-the-art of different types of workers in relation to PLPA is described in this section.

### 3.1. Human Workers and PLPA

Human acquisition of psychomotor skills involves demonstration, observation, imitation, practice, and feedback [47,48,49]. For example, aircraft painters learn how to paint aircraft by painting aircraft and getting feedback about their painting [50]. Amount, timing and quality of practice and feedback influence the level of psychomotor skill that is developed [51,52]. However, if innate psychomotor ability is very low, the acquisition of high levels of psychomotor skills can be almost impossible [53,54].

As humans develop skills, they develop neural templates for autonomous psychomotor action. These templates provide flexible patterns, which have fixed elements, variable elements, and include associative links to other templates [41]. These neural templates lead human experts to apply psychomotor skills with little, if any, conscious thought during familiar work. In addition, those with autonomous psychomotor skills can improvise in order to address new challenges in work settings [40]. Such improvisation can involve some conscious reflection about how their repertoires of psychomotor skills can be perfected to achieve better outcomes in different settings. Thus, neural templates for autonomous psychomotor action can be adapted and can have generative capacity [41]. However, as summarized in Equations (3)–(5), reflection about psychomotor skills is very different to conscious attention to physical motions, which interrupts and impairs the flow of autonomous psychomotor skills [30,31,32,33,34]. 

Instruction for a wide variety of human psychomotor skills for workplaces was formalized by medieval craft guilds [55]. Acquisition of craft psychomotor skills can involve low capital investment costs, for example, hand tools, but long acquisition times, for example, during an apprenticeship. Performance of craft psychomotor skills is characterized by high versatility but low productivity and consistency. Poor productivity and consistency of craft skills have led to many efforts to replace them with industrial practices, which began in the late 19th Century [56,57]. These include standardization of outputs and workplaces, task analysis, job design, and statistical process control [58,59]. Compared to craft practices, acquisition of industrial psychomotor skills can involve high capital investment costs, for example, an assembly line, but short acquisition time, for example, short assembly line training. Then, performance of industrial psychomotor skills is characterized by low versatility but high productivity and consistency [60].

Short acquisition times for high psychomotor productivity and consistency in industry are achieved through industrial engineering that involves optimizing work settings, rationalizing work compositions, and minimizing work uncertainty. By contrast, craft training is focused on developing the worker to deal with irregular work settings, varying work compositions and much work uncertainty, for example, at construction sites [60,61]. Accordingly, both craft and industrial workers can achieve PLPA for supervision, thought and motion in familiar settings. Industrial workers do not have the adaptive expertise that enables them to transfer skills to new settings with little, if any, conscious thought. Rather, their transfer skills are expanded step-by-step within the limitations of one type of production organization, such as consumer electronics manufacturer or car maker, through job enlargement [62].

### 3.2. Cyborg Workers and PLPA

Humans can be described as cyborgs when their natural capabilities are restored or enhanced by the addition of technologies [25,63,64,65]. Humans who are enhanced by technologies can already have autonomous psychomotor work skills. A wide range of technologies can enhance their abilities and skills. Some technologies can be implanted permanently [66,67], while others can be worn persistently [68,69].

Some enhancing technologies do not depend upon external energy supply for their operation. For example, non-motorized exoskeletons can be strapped on like a harness to enhance human strength and endurance. In particular, such exoskeletons can have carbon fiber rods that act as artificial tendons, which bend and straighten to support the wearer’s movement. These have been reported to be beneficial for work performance and are easy to wear throughout the working day [70]. Other enhancing technologies can be motorized adaptations of well-established work wear. For example, there are motorized gloves that improve human grasping through combinations of artificial sensors, actuators, and tendons. Humans are well used to wearing work gloves, and these motorized gloves reduce the manual force needing to be exerted by the human wearer [71].

Technologies that can restore or enhance natural capabilities in the workplace also include various types of wearable robotics, such as motorized exoskeletons, orthoses, and prosthetics [72]. Wearable robotics can enhance abilities that contribute to psychomotor work skills such as strength and endurance. However, use of wearable robotics can require conscious thought, which impairs autonomous performance of psychomotor skills [34,35]. Getting into the flow of autonomous performance of psychomotor skills can be hindered by difficulties of achieving sensory fusion between the human and the wearable robotics [73,74]. For example, it may not be possible to achieve autonomous performance of psychomotor skills by controlling wearable robotics through via brain-machine interface systems (BMI). This is because physical movement in performance of a psychomotor skill can begin before related brain waves start [75,76].

The acquisition and deployment of cyborg psychomotor skills can involve craft practices and industrial practices. With regard to craft practices, there can be novel challenges in practice and feedback such as learning how to control exoskeletons via brain–machine interface (BMI) systems [77]. Intrinsic feedback about results can be provided by the end condition of the work task in much the same way as for human psychomotor work skills. For example, a bent nail provides intrinsic feedback that the work task has not be completed successfully, whereas, a sunk nail provides intrinsic feedback that the work task of hammering in the nail has been completed successfully. However, feedback about performance can be more difficult, because sensing the performance of movement can be obscured by the wearing of exoskeletons, etc. [78]. Feedback about performance is particularly important for safety-critical tasks, where unsuccessful results can have grave consequences [79].

When cyborgs are employed in typical industrial settings such as factories, typical industrial engineering practices can be applied to optimize work settings, rationalize work compositions, and minimize work uncertainty [80]. However, industrial engineering practices, such as job design and systems engineering, are not so obviously applicable to the work of cyborgs in other settings [81]. In terms of trade-offs, acquisition costs and times for cyborg psychomotor work skills can be higher than for humans. However, increased strength and endurance could increase performance productivity and consistency.

Overall, the use of enhancing technologies can make PLPA more difficult to achieve. This is because of the need to progress beyond conscious thought about enhancing technologies when using them in the performance of psychomotor skills. Potential for autonomous performance of psychomotor skills could be increased by improvements to the ergonomics of wearable robotics that reduce the amount of conscious thought needed in their use [82,83]. Over the longer term, it may be possible for neural plasticity to bring adaptation of relevant brain functioning if a wearable robot is used for long enough. This could facilitate autonomous performance of psychomotor skills with the wearable robotics. Examples of such effects from neural plasticity are provided by people who develop autonomous understanding of inputs received via Cochlear Implants [84,85]. However, wearable robotics can involve many brain areas, and so neural adaptation may take longer or may not be possible. Hence, as summarized in Equations (3)–(5), the flow of autonomous action can be interrupted by conscious thought brought about by humans thinking about the enhancing technologies that they are implanted with or wearing.

### 3.3. Robot Workers and PLPA

Psychomotor work skill acquisition costs and times for robots rise in accordance with the sophistication and variation of skills that are acquired. The acquisition of any psychomotor work skills by robots is a profound challenge [86,87]. The acquisition and deployment of robot psychomotor skills can involve craft practices and industrial practices. For example, robot apprenticeship learning [88] and robot learning from demonstration [89] draw upon craft practices. Accordingly, they can involve demonstration, observation, imitation, practice, and feedback [90,91]. A target for robot learning from demonstration is to enable adaptation of skills to new settings with minimal extra programming [89,92,93]. At the same time, techniques similar to industrial task analysis and job design can be applied. These include determining which aspects of demonstrations are essential [94,95], and how demonstrations can be imitated by robots [96,97,98]. 

Overall, industrial engineering practices to optimize work settings, rationalize work compositions, and minimize work uncertainty are well-established in the industrial deployment of robots. Also, there can be some concurrent development of the robot worker and the work when standard robots are programmed for the specific requirements of particular tasks. However, industrial practices such as standardization of workplaces and outputs are not so obviously applicable to all robots outside of factory settings [99]. Furthermore, robot workers are far from achieving the same level of least action as human workers in autonomous psychomotor work skills. In particular, unlike humans, robots do not have embodied general psychomotor abilities that can provide foundations for flow states in more specific psychomotor work skills. Rather, soft robotics are at an early stage of development where only a few rudimentary psychomotor capabilities can be encoded within the physical materials of robots or enabled through smart materials that respond automatically to sensory inputs. Also, morphological computation, which involves the robot body contributing to overall orchestration of intelligent robot behavior, is at an early stage of development. The combination of soft robotics and morphological computing is analogous with structuring of human psychomotor functioning where morphology facilitates perception and control, and so may eventually facilitate robot conformance to PLPA [100,101,102,103]. However, the transfer of psychomotor skills to new settings is an unresolved challenge, which limits the potential of robots to function without supervision in heterogeneous work [104,105,106,107]. For example, this is leading Toyota to invest in combining skilled human workers with robot assistants rather than increasing robotic automation of vehicle production [108]. Thus, as expressed in Equation (4), robot workers are more likely to have side information, for example, from external supervision to process than human workers. Hence, internal action, as expressed in Equation (5) is likely to involve greater complexity.

### 3.4. Comparison of the PLPA Potential of Human, Cyborg and Robot Workers

A summary of worker types in relation to PLPA is provided in Table 1. This shows that human workers can have the most autonomous psychomotor work skills and robot workers fewer autonomous psychomotor work skills. In between, cyborg workers are hindered by the extra conscious thought required to carry out psychomotor work skills with exoskeletons, etc.

However, there are often shortages of human autonomous psychomotor skills [21,22]. This can be because of, for example, insufficient human trainers being available to provide demonstration and feedback. This has led to development of alternatives for providing training in psychomotor skills: for example, mixed reality training systems that incorporate haptic feedback [79]. Yet, skill shortages persist [109]. Moreover, development of robots that can carry out psychomotor skills autonomously is of vital importance. This is because, for example, there are locations where application of psychomotor skills are extremely dangerous for humans. In addition, there are many types of psychomotor work that are repetitive and otherwise arduous, which lead to chronic human health problems. Accordingly, different types of workers may often need to be combined in hybrid intelligence systems [26]. As explained in more detail in the next Section 4, the contribution of PLPA is to focus attention during the engineering design of autonomous systems on the inherent advantages of minimizing both internal actions (e.g., minimal conscious thought and minimal computation effort) and external actions (e.g., minimal biomechanical motion and minimal mechatronic motion). In particular, least action is provided by the engineering design that offers minimal internal action and minimal external action compared to all other designs.

## 4. Work: Engineering Work Setting, Composition and Uncertainty towards PLPA

### 4.1. Engineering Design Rules and Strategies for PLPA

**Figure 3:**
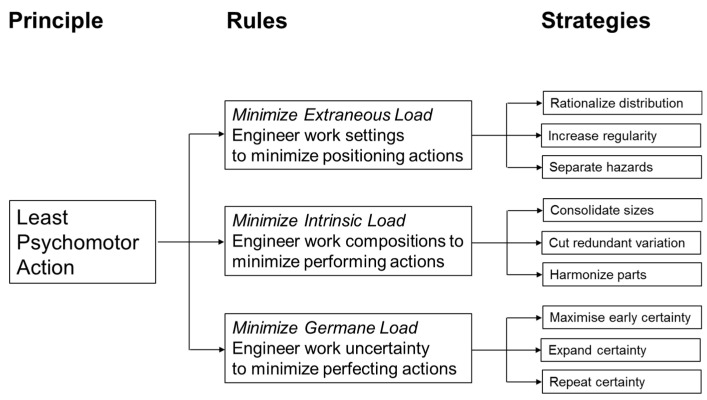
PLPA in relation to rules and strategies for engineering design of work.

In this section, opportunities for engineering design towards PLPA are explained in relation to work setting, work composition, and work uncertainty [46]. In terms of cognitive/computational loading, work setting relates to extraneous load; work composition relates to intrinsic load, and work uncertainty relates to germane load. Extraneous load includes load that is not intrinsic to a work task, but nonetheless must be addressed to carry out the work task. Intrinsic cognitive load arises from fundamental characteristics of a work task. Germane cognitive load can be related to the work put into creating a permanent store/schema for the work task. These categorizations are derived from the widely applied Cognitive Load Theory, and its previous application in modelling situated entropy in factories [110]. In the following cases, the modelling of situated entropy is extended beyond the previous cases [110]. In particular, progress towards achievement of PLPA is expressed in terms of reduction of situated entropy involved in addressing distribution, regularity and hazard of work settings; size, variability and number in work composition; timing, extent, and repetition of work uncertainty. It is important to note that simple principles, such as that provided by PLPA, can lead to many simplifications of otherwise complicated work. These simplifications can lead to radical performance improvements throughout set-up and operation. As summarized in Figure 3, a simple principle, such as PLPA, can provide unifying objectives for engineering design rules that can be realized through following supporting engineering design strategies.

This combination of principle, rules and strategies has proven to be highly effective in the realization of other objectives, which are now well-established, but were previously seldom recognized [111,112]. Engineering of work setting, work composition and work uncertainty can be considered in terms of pragmatic interaction between work and worker. Pragmatics is concerned with the ways in which context contributes to the construction of meaning. In terms of pragmatic interaction between work and worker, the physical aspects of work can carry new context that bring new Information Gain but potentially also new sources of situated entropy. Hence, engineering of work should be focused upon achieving *Net* Information Gain [110].

### 4.2. Positioning Action—Work Setting (Extraneous Load)

As in the past, what combination of workers enables least action to achieve a work goal continues to be influenced by the work setting [113,114]. In particular, the work setting affects the action required to get into position to perform psychomotor skill. If work can be designed so that work pieces can be always brought to fixed points, then workers only have to position themselves once and positioning action is minimal irrespective of the type of worker. However, static work positions cause musculoskeletal disorders for human workers. Accordingly, human workers should reposition themselves frequently for health reasons [115]. Zero positioning action is also not a healthy option for cyborg workers. Indeed, a potential risk of technologies for enhancing strength and endurance is that they could lead to the human wearer feeling comfortable in static positions for longer. Further, being in static positions for longer can cause disorders in the body’s wide fascial network of fibrous tissues [116]. Thus, only robot workers are suitable for zero positioning action. Hence, if work can be designed so that work pieces can always be brought to fixed points, the positions of robots should be fixed to enable zero positioning action.

However, fixed position and zero positioning action is not technically feasible in most work. Indeed, it has been argued that the ability to explore unknown environments is a prerequisite for a truly autonomous robot [2]. Positioning actions can be influenced by the characteristics of work settings, including their distribution, regularity, and hazard. An example is agricultural work that is distributed across different positions, which have different irregularities in weather conditions and ground conditions that bring different levels of hazard [117,118].

Workers typically react to sensory inputs when positioning themselves for a task. As already mentioned in Section 2, Friston’s information-based model [43,44,45] can be drawn upon when describing quantitatively positioning aspects of psychomotor work. For example, suppose that a worker takes position *x* after reception of sensory input *r* under a model *w* of the world. Then, the informational load of selecting a position is measured with conditional entropy

(7) $$H\left(X)=H(R|W\right)=-\iint_{r,w}^{}p\left(r|w\right)p\left(w\right)\log_{2}p\left(r|w\right)drdw.$$

In words, conditional entropy *H*(*R*|*W*) represents an average “surprise” of receiving unexpected sensory input in an otherwise known environment. Intuitively, to reduce part of the internal action *S_i_* corresponding to conditional entropy *H*(*R*|*W*), a working environment should be designed in such a way that “surprises” are minimized by, for example, avoiding hazards and increasing regularity.

### 4.3. Performing Action—Work Composition (Intrinsic Load)

What combination of human and non-human workers enables least action to achieve a work goal is influenced by work composition [119,120]. In particular, work composition affects the action required to perform psychomotor skill. Work composition includes the size, variability, and number of parts. If parts are massive or if parts are tiny, handling equipment is needed. The extent to which any type of worker is needed is influenced by the variability of parts. If parts never vary in their properties, then investment in an autonomous handling system is technically feasible. If there many parts that are always the same, then investment in an autonomous mechanical handling system is economically viable. Hence, if there is a high number of invariant parts of extreme size, no workers are needed. An example is the use of Intelligent Autonomous Vehicles (IAVs) to handle shipping containers at seaports [121]. However, having no workers is not technically feasible for most work. Rather, work composition affects the performing of psychomotor skills by different types of workers.

Least psychomotor action for human workers is facilitated by parts that are about the grip size of the human hand. Variability does not necessarily increase psychomotor action. For example, the shape and sizes of apples vary, but seldom enough to involve notable increases in psychomotor action during apple harvesting. Action can be reduced if a large number of smaller parts are consolidated into a smaller number of larger parts. However, action can increase if several human workers are needed to handle one part. To avoid this, a wide variety of equipment has been developed to bring mechanical strength to the aid of human workers, including Intelligent Assist Devices (IAD) [122]. Enhancing technologies that enable human workers to become cyborg workers can reduce action required in handling work pieces that are above the human optimum, especially if they reduce the need for IADs. Robot workers can have greater potential to handle large work pieces. However, there are limits to what any worker can handle alone. For example, fitting a long heavy multi-feature dashboard assembly into an automotive vehicle from one installation position requires specialized IAD to support the worker [123]. 

For example, suppose that a worker can be in one of possible positions *X* and that a given task can be completed in one of possible ways *Y*. Let us denote the current position by *x* and assume that the worker selects position *x* with probability *p*(*x*). Furthermore, let the worker select a certain way to complete the task, denoted by *y*, with probability *p*(*y|x*). Then, the complexity of performing a given task is measured with the conditional entropy

(8) $$H\left(Y|X\right)=-\iint_{x,y}^{}p\left(x\right)p\left(y|x\right)\log_{2}p\left(y|x\right)dxdy.$$

The impact of work composition on performance of workers can be modeled as follows. Let us assume that a work composition is such that it provides side information denoted by *u* with probability *p*(*u|x*) Then, the conditional entropy *H*(*Y*|*X*) is reduced to conditional entropy of *Y* given both *X* and *U*, or,

(9)  H(Y|X,U)=H(Y|X)−I(Y,U|X)≤H(Y|X) 

with equality if and only if *Y* and *U* are independent given *X*. The symbol

(10) $$\;I\left(Y,U|X\right)={∭}_{x,y,u}^{}p\left(x\right)p\left(y,u|x\right)\log_{2}\frac{p\left(y,u|x\right)}{p\left(y|x\right)p\left(u|x\right)}dudydx\;$$

denotes mutual information between random variables *Y* and *U* given *X*. In other words, the situated entropy of performing a task is reduced when additional information is provided, for example, parts are designed to fit in only one way, a product is assembled using modules, etc. Intuitively, when additional information is provided, the number of possible ways to complete a given task is significantly reduced. Consequently, the task complexity is reduced and situated entropy is reduced. However, processing that additional information may incur additional energy cost that needs to be taken into account.

### 4.4. Perfecting Action—Work Uncertainty (Germane Load)

What combination of human and non-human workers enables least action to achieve a work goal is influenced by work uncertainty. In particular, the timing, extent and repetition of work uncertainty affects action required to perfect psychomotor skill. If all the work to be undertaken is always certain from the outset, then all can be perfected at the outset: for example, in so-called “lights out” factories where all work is carried out by autonomous robot workers [124]. By contrast, if work composition is not certain until work is completed, then workers that have adaptive expertise are needed. If the whole extent of work is uncertain, then many workers who have adaptive expertise are needed. Subsequently, if there is little, or no, repetition of post-completion work certainty, then workers with adaptive expertise are needed always. An example is work involved in building renovations where original building drawings are not available and original construction has been overlaid by several past refurbishments [7,9].

Most work involves at least some uncertainty and needs workers with at least some adaptive expertise. Hence, the widespread interest in developing autonomous robots that can deal with non-routine work. However, frequent action to perfect psychomotor work skills is required for adaptive expertise. For example, human workers reflect about how their repertoires of psychomotor skills can be perfected to achieve better outcomes in different settings. This involves the adaptation of neural templates [40,41,42]. For cyborg workers to perfect psychomotor skills in new settings, there are the requirements for action needed by human workers, plus the requirement for action involved in learning how to apply enhancing technologies in new settings. For robot workers, transfer learning required for adaptive expertise also involves action. A particular problem for robot transfer learning can be the low degree of generalization of experimental results achieved at single locations such as university labs [104]. By contrast, the general psychomotor abilities of humans, which provide foundations for more specific psychomotor work skills, are developed in diverse locations throughout years of growing up. In the terminology of robot transfer learning, this means that humans have many source domains for any one new target domain, but robots may have only one source domain for many new target domains [106].

As already mentioned in Section 2, Friston’s information-based model [43,44,45] can be drawn upon to describe quantitatively perfecting aspects of psychomotor work. For example, suppose that a worker receives sensory input *r* as the result of his actions during performing a task. We assume that *v* is an unknown quantity that caused the sensory state *r* and denotes the true distribution of the causes by *q*(*v*|*r*). The worker tries to infer possible cause *v* for the sensory state *r* using her/his probabilistic representation of the world μ. The so-called recognition density *p*(*v*|*μ*) describes to the worker’s probabilistic representation of the causes of the sensory inputs. Then, the informational load of perfecting actions is measured with relative entropy

(11) $$H\left(Z|X,Y)=D(p||q\right)=\int_{v}^{}p\left(v|\mu \right)\log_{2}\frac{p\left(v|\mu \right)}{q\left(v|r\right)}dv.$$

In words, relative entropy *D*(*p*||*q*) describes how similar probability distributions *p* and *q* are, that is, how well the worker perceives and understands the process of production. The worker can minimize the part of internal action *S_i_* corresponding to perfecting actions by minimizing the relative entropy term *D*(*p*||*q*). This task is usually accomplished by active inference, that is, by optimizing perception and adapting worker’s recognition density *q*(*v*|*r*) into a better approximation of the true distribution *p*(*v*|*μ*).

## 5. PLPA Modelling Examples

### 5.1. PLPA Modelling

In this section, three PLPA modelling examples are provided where there are limited opportunities to implement the PLPA engineering strategies summarized in Figure 3. In other words, these are all challenging cases for psychomotor work. PLPA modelling can begin with modelling a psychomotor work task’s situated entropy in terms of positioning *X*, performing *Y* or perfecting *Z* (Equations (7)–(11)). *H(X,Y,Z)* the total situated entropy involved in positioning, performing and perfecting (Equation 6) is a determining input into the modelling of internal action *S_i_* (Equations (3)–(5)), which is added to the external action *S_e_* (Equation (2)) in order to determine total action *S* (Equation (1)).

### 5.2. Positioning Action Example: Extraneous Load from Agricultural Work Settings

Agricultural work is carried out at wide variety of work settings, including low, medium and high altitudes where ground can be flat, sloping or steep. Robot workers are best suited to low altitudes with flat ground where they can more easily be brought into, and remain stable, in work positions. Conversely, they are less suited to high altitudes with steep ground [117,118]. The relative potential for minimal positioning actions *X* by robot workers at different altitudes with different ground conditions can be modelled through application of Equation (7): where conditional entropy *H(X) = H*(*R*|*W*) represents the average surprise of receiving unexpected sensory input *r* whilst in an otherwise known work setting for which the worker has a model *w*. For the purposes of this example that is focused on extraneous load from work setting, it is assumed that performing actions *Y* and perfecting actions *Z* are equal for robot, cyborg and human workers. Consequently, internal actions *S_i_* (Equation (3)) are most influenced by positioning actions. Also, external actions *S_e_* (Equation (2)) are principally dependent on external positioning actions *X* as external performing actions *Y* and external perfecting actions *Z* are assumed to be the same for different types of workers. Hence, for the purposes of this example, different work types’ total psychomotor action *S* (Equation (1)) is most dependent upon different work types’ positioning actions.

Consider, for example, sloping work sites where it is not economically viable to engineer out extraneous load by hardscaping the ground. In such situations, the extraneous load introduced by ground conditions increases as the ground becomes more slippery and undulating from combinations of heavy rainfall and worker traffic. In this situation of slippery undulating ground, all types of workers go into a “choke” state as shown in Figure 2 as both internal action and external action are focused upon recovering stable work position. The bigger the average surprise from *r* as the worker slides and slips on the wet sloping ground, the bigger is *H(X)* and as a result the bigger is total action *S*.

Average surprise depends upon *r* and *w*. For example, the human worker has general psychomotor abilities based on neural templates that are developed in diverse situation conditions throughout years of growing up. Human templates provide flexible patterns, which have fixed elements, variable elements, and include associative links to other templates. Thus, if *r* exceeds the boundaries of *w* provided by one neural template the human worker can refer to the *w* of a more appropriate neural template. For example, if the human worker slips and falls to the ground, the human worker can refer to the relevant *w* developed throughout years of childhood falling down and getting up while playing games outside. Hence, falling down is a discrete surprise that is followed by referring to the *w* for getting up from having fallen down. Against this *w*, sensor inputs *r* provide low average surprise as the human worker gets up from having fallen down [41]. Thus, the extraneous embodied cognitive load is momentarily high, but then is reduced by reference to the relevant *w*. For the cyborg worker wearing an exoskeleton, the congruence of sensory inputs *r* to *w* for getting up from a falling position is confounded by the additional surprise from *r* due to misalignments between the human body and the exoskeleton framework. For the robot worker, it is possible to have *w* for upright working and another *w* for recovering from falling. The sensory trigger for switching between them being as simple as sudden reduction of the proximity of top of robot to the ground. However, although robot *S_i_* is relatively low, robot *S_e_* is relatively high because of the multiple mechatronic actions needed to reestablish an upright position on slippery sloping ground, where the use of mechanical claws etc., can be counterproductive as they churn up the soft ground even more. *S_e_* is increased further by the additional external actions involved in human and cyborg workers assisting the robot worker to reestablish an upright position.

Thus, PLPA is realized by human workers in the situation of slippery undulating sloping ground at medium altitudes. This is because *S_i_* is lowest by human reference to appropriate *w* for human general psychomotor abilities and *S_e_* is lowest because only human body weight is being maneuvered and it is maneuvered using human general psychomotor abilities.

### 5.3. Performing Action Example: Intrinsic Load from Soft Products Work Composition

Soft products, such as clothing and furnishings, are composed of far fewer components than metal products such as cars. However, soft products comprise soft materials such as cloth, which unlike metals, bring high intrinsic load due to deformation into unpredictable geometries during manufacturing operations. The relative potential for minimal performing actions *Y* by robot workers in apparel production can be modelled through applications of Equations (8)–(10) where conditional entropy *H(Y|X)* represents the complexity of performing a task in position *X*. For the purposes of this example that is focused on intrinsic load from work composition, it is assumed that positioning actions *X* and perfecting actions *Z* are equal for robot, cyborg and human workers. Consequently, internal actions *S_i_* (Equation (3)) are most influenced by performing actions *Y*. Also, external actions *S_e_* (Equation (2)) are principally dependent on external performing actions *Y* as external positioning actions *X* and external perfecting actions *Z* are the same for the different types of workers. Hence, for the purposes of this example, different work types’ total psychomotor action *S* (Equation (1)) is most dependent upon different work types’ performing actions *Y*.

As expressed in Equation (10) as *I(Y*,*U|X)*, the effectiveness of new side information *I* from the engineering of work compositions depends upon the extent of mutual information between *Y* and *U* given *X* where *U* represents instances of *I*. As expressed in Equation (9) as *H(Y|X*,*U)*, the conditional entropy *H(Y|X)* expressed in Equation (8) is reduced to conditional entropy of *Y* given both *X* and *U*, but if and only if *Y* and *U* are independent given *X*. However, modelling of *Net* Information Gain recognizes that introduction of new side information *I* through instances *u* may also introduce new sources of situated entropy *c_H_(t)*, and/or additional sources of energy consumption *c_I_(t)* in *S_i_* (Equation (5)).

One innovation to enable increased robot workers in the manufacturing of soft products is improved robot CAD/CAM (Computer-Aided Design/Computer-Aided Manufacturing). This involves manufacturing instructions being incorporated into CAD files that are transferred to the robot to guide its manufacturing actions, which include advanced machine vision and actuators for tracking and correcting distortions in cloth during manufacturing. For example, micromanipulators powered by precise linear actuators are deployed to correct cloth distortions with submillimeter precision. An unrelated method to this CAD/CAM innovation involves using chemicals to change temporarily the properties of cloth and so reduce its intrinsic load for manufacturing actions. This is congruent with the PLPA engineering strategy of cutting redundant variation. It is done by drenching cloth in a liquid thermoplastic solution that makes cloth so stiff that it no longer undergoes unpredictable distortions during manufacturing. Robots can then sew and shape the stiff cloth without the need for advances in CAD/CAM. Subsequently, the cloth in the completed apparel is washed with warm water and becomes soft once again [125].

The CAD/CAM method does not directly reduce the intrinsic load of work composition. Rather, it increases *I* by CAD/CAM transfer from designer to robot without additional situated entropy *c_H_(t)* and low additional sources of energy consumption *c_I_(t)*. However, the millions of instances of *u* from millions of iterations of machine vision and actuating bring high *c_I_(t),* which can increase situated entropy *c_H_(t)* if machine vision cannot accurately differentiate distortions in cloth, for example, due to unusual color patterns. By contrast, the chemical method reduces situated entropy *c_H_(t)* throughout manufacturing without the need for millions of instances of *u*. However, *S_e_* is increased by the need to drench the manufacturing cloth and to wash the manufactured soft product. Thus, *S_i_* is higher for the CAD/CAM method but *S_e_* is higher for the chemical method. Yet, both of these methods can be compared favorably to established labor intensive production methods where human teams perform hundreds of actions to make just one jacket. The introduction of exoskeletons and other cyborg technologies, such as powered gloves, can do little to improve the productivity of labor-intensive methods in soft production manufacturing. This is because such manufacturing typically involves lightweight materials and very precise hand-to-eye coordination in use of fine motor skills rather than the heavy weight work and gross motor skills that can be better enabled by cyborg technologies. Thus, PLPA is most likely to be realized by innovations in robotics that can minimize both *S_i_* and *S_e_* rather than reducing one but increasing the other. Nonetheless, finishing work should be left to human hands where the market prices of soft products are increased by their being handmade to some extent.

### 5.4. Perfecting Action Example: Germane Load from Construction Work Uncertainty

In the construction industry, there are limited opportunities to implement the PLPA rule of engineering uncertainty out of work. This is because much of construction work is characterized by uncertainty, for example, because different building clients have different requirements for different types of buildings at different building locations. The relative potential for minimal perfecting actions *Z* by robot workers in construction work can be modelled through application of Equation (11), where germane load of perfecting actions *H(Z|X*,*Y)* is considered in terms of relative entropy *D(p||q)* that describes how similar probability distributions *p* and *q* are. In particular, how similar are true distribution of causes *q(v|r)* and recognition density *p(v|μ)*, in other words, how well the worker perceives and understands the process of production. For the purposes of this example that is focused on germane load from work uncertainty, it is assumed that positioning actions *X* and performing actions *Y* are equal for robot, cyborg and human workers. Consequently, internal actions *S_i_* (Equation (3)) are most influenced by perfecting actions. Also, external actions *S_e_* (Equation (2)) are dependent principally on external perfecting actions *Z* as external positioning actions *X* and external performing actions *Y* are the same for different types of workers. Hence, for the purposes of this example, different work types’ total psychomotor action *S* (Equation (1)) is dependent most upon different work types’ perfecting actions, which involve active inference in order to adapt worker’s recognition density *q(v|r)* into a better approximation of the true distribution *p(v|μ)*.

As summarized in Figure 2, active inference interrupts the flow of autonomous actions and leads to choke state for both *S_i_* and *S_e_* as the worker stops action to try to infer what action to select next. Active inference is triggered when an unknown quantity *v* causes sensory input *r*. Active inference involves the worker trying to determine possible cause *v* for the sensory state *r* using its/her/his probabilistic representation of the world *u*. Germane load from creating an updated store/schema of production knowledge involves a succession of additional *S_i_* and *S_e_* including exploring extant internal knowledge schema, seeking new external information *I*, and adding new information to extant knowledge schema. Seeking new information *I* can involve multiple iterations of trial and error to try to discover what works and/or seeking out external supervision.

For the robot worker, trial and error has limited potential to enable robot learning amidst the sparse learning rewards provided by the continual uncertainty of construction work. In particular, the robot worker’s probabilistic representation of the world *u* cannot ascend to the level of complete bespoke assemblies that are included in building construction, such as outside walling and inside furnishings with one-of-a-kind geometries. Rather, the robot worker’s *u* can be reinforced through robot learning up to the level of parts and tools. For example, the robot worker can learn that different sizes of cutting tools are used to remove different sizes of surplus materials in fitting work. For example, an electric saw, an electric planer and an electric sander are used to remove centimeters, millimeters and micrometers, respectively. For example, the robot worker can learn that a screw is fixed with a screwdriver and that a specific size of screw head is fixed with a specific size of screwdriver bit. In addition, the robot worker can learn where to use what size of screw, because that is closely related to the thickness of what is to be fixed with the screw. By contrast, fitting and fixing a one-of-a-kind inside furnishing, such as reception desk, up against the one-of-a-kind geometry of the inside surface of a one-of-a-kind outside wall requires a higher level of *u*, which incorporates how different cutting and fixing tools can be used in situation-specific combinations to fit and fix the one-of-a-kind to the one-of-a-kind. Thus, relative entropy *D(p||q)* is high for the robot worker when there is a disconnect between probability distributions *p* and *q* when one-of-a-kind true distribution of causes *q(v|r)* is at a higher level than the robots recognition density *p(v|μ)*. In such situations, both *S_i_* and *S_e_* can be minimized by the robot being programmed to immediately send for external supervision and shut down all actions until external supervision is provided.

For human workers, establishing knowledge of exactly what to do in one-of-a-kind situations involves improvisational inference. This includes trial and error with the aim of moving forward a little and learning something along the way. Thus, relative entropy *D(p||q)* is reduced as improvisational inference concurrently changes both true distribution of causes *q(v|r)* and recognition density *p*(*v*|*μ*) [40,42,126]. However, the number of iterations of trial and error required and so the amount of unproductive *S_i_* and *S_e_* involved to reduce relative entropy *D(p||q)* is influenced by the extent of the human worker’s existing production knowledge schema and the extent of the human worker’s endurance. Both of these limitations can be addressed through implementation of cyborg technologies. In particular, technologies such as augmented reality (AR) can facilitate retrieval of relevant information from external knowledge bases, while the wearing of exoskeletons can increase endurance when some trail and error is needed is needed in a variety of work situations throughout the working day. Thus, PLPA is realized by cyborg workers in the situation of fitting and fixing one-of-a-kind to one-of-a-kind. This is because *S_i_* is lowest by reference to appropriate *I* through AR and *S_e_* is lowest because physical motion is supported by exoskeletons. However, the realization of PLPA by cyborg workers depends cyborg technologies not introducing additional sources of situated entropy *c_H_(t)* and additional sources of energy consumption *c_I_(t)*, which exceed the those brought about by the limitations of human workers’ schema and endurance [110].

### 5.5. Summary

A summary of the three examples is provided in Table 2. This shows that different types of workers can be best suited to realizing PLPA in different situations. Accordingly, hybrid intelligence production systems are needed that deploy different types of workers in different situations within overall production processes.

## 6. Discussion

### 6.1. Background

**Figure 4:**
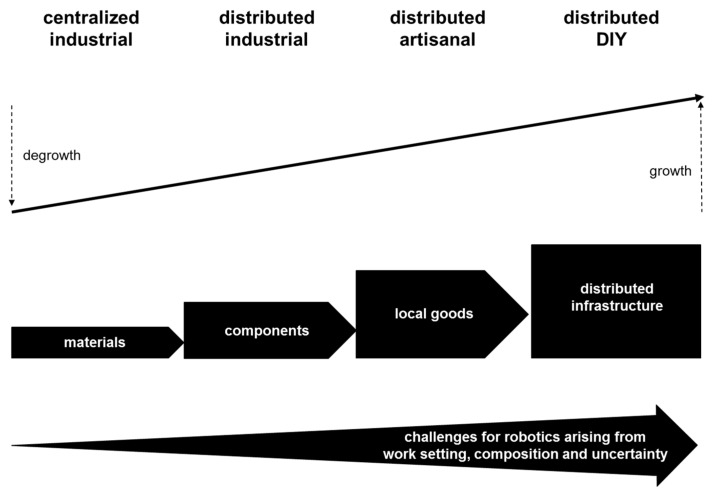
Increasing geographical and social distribution of production.

Hype about advances in robotics has led to widespread debate about robots undertaking all work [17,18,19]. However, studies indicate that there is much work where robots workers add most value when they work together with human workers [20,21]. Moreover, potential for robots to take over all psychomotor work is limited by the huge diversity of needs for psychomotor skills around the world [22,23,24]. In particular, as summarized in Figure 4, centralized industrial production is decreasing in many parts of the world while distributed artisanal and do-it-yourself (DIY) production is increasing. This new continuum from degrowth of centralized industrial production to growth of distributed production is enabled by advances in distributed production technologies including moveable factories and desktop hybrid manufacturing machines [127,128,129,130]. Increasing geographical and social distribution of production leads to increasing diversity of work settings, work composition and work uncertainty, which increases the challenges for implementation of full automation with robotics. Hence, there is greater need for hybrid intelligence systems that deploy humans, cyborgs and robots in manual work. Different types of psychomotor skills are needed in distributed artisanal production and distributed DIY production. For example, finishing work by human hands can attract most market value in artisanal production, such as hand-finished jackets and shoes. By contrast, heavier work in distributed DIY production, such as the erecting solar arrays for local energy generation, can benefit from cyborg technologies for increasing human strength and endurance. Meanwhile, robotics can be deployed in the production of materials and components that go into hand finished local goods and distributed infrastructure. Thus, while the overall need for psychomotor skills increases, there is considerable variation in the level of finesse needed in different types of distributed production work. 

### 6.2. Implications for Theory Building

Hitherto, debate about what type of worker should undertake physical work has lacked the specificity that can be provided by modelling based on theoretical foundations relevant to modelling situated entropy. For example, some of extant debate involves speculation that autonomous robots will bring “hollowing out” of middle-income employment. In particular, it is argued that autonomous robots can easily replicate and replace jobs that require relatively recently evolved human skills, such as logic, which feature in middle-income jobs. Conversely, it is argued that jobs which robots cannot easily replicate are those that rely on the deeply evolved skills like mobility, which feature in lower-income jobs [87,131]. However, such arguments are flawed if they involve assumptions that there is inevitable separation between middle-income mental work and low-income physical work. For example, income analyses indicate that plumbers can have annual disposable incomes that are similar to those of upper middle-income jobs in professions such as medicine [132]. Moreover, low-income manual work jobs do not have to remain low-income jobs. Instead, income can be increased if a human becomes a cyborg worker and works together with robot workers in a hybrid intelligence system. This is possible when there is concurrent development of workers and work to facilitate PLPA.

The modelling in this paper is based on a conceptual framework that brings together three theoretical themes relevant to situated entropy in physical work: embodied cognitive load, workplace pragmatics [110], and the Principle of Least Action [26,27,29,30,31]. The preceding theory-based conceptual framework reported earlier in Entropy [110] has been expanded in three respects. First, through detailed consideration of the Principle of Least Action [26,27,29,30,31], which is expressed in relation to psychomotor work here as the Principle of Least Psychomotor Action. Second, work setting, work composition and work uncertainty have been incorporated. Third, work setting has been related to extraneous load; work composition has been related to intrinsic load; and work uncertainty has been related to germane load.

### 6.3. Implications for Applied Research

Hitherto, the debate about what type of worker should undertake physical work has lacked modelling of physical work in relation to different types of workers (human, cyborg, robot). Also, the debate has not been informed by modelling three different types of Information Gain/cognitive load (intrinsic, extraneous, germane). Furthermore, the debate has not included modelling of different work cases such as agriculture and construction. Moreover, the unifying Principle of Least Action has not been considered.

For example, the capital-skill complementarity hypothesis [133,134] has been applied in efforts to determine where robot workers will be replaced by human workers and where robot workers will be used to complement human workers. In particular, it has been argued that as the cost of robot workers go down relative to the costs of human workers, robot workers will replace low skill human workers and complement high skill human workers. This pattern of replacing and complementing is anticipated in factory work where the work uncertainty is engineered out of work settings and work compositions [135]. More broadly though, it has been argued that there cannot be generalizable forecasts outside of factory production from the capital-skill complementarity hypothesis [99].

By contrast, the modelling in this paper addresses the specifics of engineering work setting, work composition and work uncertainty in particular work cases in relation to three different types of workers: human, cyborg and robot. Furthermore, the modelling addresses the need for least action in cases outside of factory production, for example, in agriculture and construction.

### 6.4. Implications for Practice

In practice, modelling PLPA can advance measurement of the Triple Bottom Line (i.e., profit, people, planet) [136]. With regard to the planet, realization of PLPA can reduce energy consumption by reducing the total number of actions involved in positioning, performing and perfecting psychomotor skills. Moreover, concurrent development of workers and work to realize PLPA can contribute to avoiding unnecessary implementations of robot workers that are driven by hype [137,138]. This is important because the fabrication of robot workers involves increased extraction of finite resources from the lithosphere, related disruption to the biosphere, and further expansion of the technosphere [139]. With regard to people, concurrent development to realise PLPA incorporates human workers and cyborg workers, as well as robot workers, in addressing the specific characteristics of particular work settings, compositions and uncertainties.

**Figure 5:**
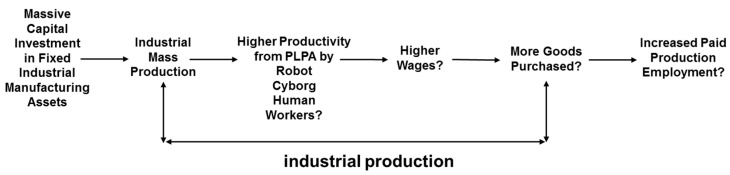
Industrial context for situated entropy PLPA modelling.

With regard to financial profit, failure to undertake concurrent development to realize PLPA can increase costs. For example, a 10 million euro investment in automating the installation of car windshields on an assembly line inadvertently increased the number of actions in windshield installation. As a result, operating costs increased [140]. This is not a one-off case. For example, global companies, such as Mercedes and Toyota, have suffered the costs of first installing robots and then having to replace some of them with human operatives [141]. The industrial financial context for modelling the comparative potential for different types of worker to achieve higher productivity through realization of PLPA is summarized in Figure 5; in particular, modelling how humans will get wages to maintain the economic growth cycle of higher production wages leading to purchasing of more goods leading to more industrial production leading to increased production employment [142].

**Figure 6:**
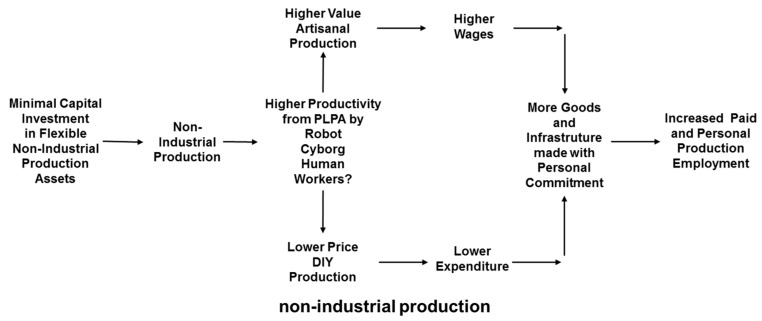
Non-industrial context for situated entropy PLPA modelling.

The non-industrial financial context for modelling of the comparative potential for different types of worker to achieve higher productivity through realization of PLPA is summarized in Figure 6. In particular, this shows that artisanal work can be focused on higher value production while DIY can be focused on lower cost prosumption involving people consuming the outputs of their own production work [143].

## 7. Conclusions

A Principle of Least Psychomotor Action (PLPA) has been introduced as follows: the preferred combination of workers is the combination of worker types that can carry out psychomotor work with the least internal action and least external action. PLPA encompasses three types of workers: human, cyborg, and robot. PLPA encompasses positioning, performing and perfecting psychomotor skills work. Through introduction of engineering design strategies and modelling of situated entropy, these have been related to the development of the work setting, work composition, work uncertainty, and concurrent development of worker and work. PLPA is founded upon the Principle of Least Action. Modelling of related situated entropy is informed by previous studies by others concerned with connections between information theory and thermodynamics.

When considering directions for future research, there are many types of work that require combining different types of workers. These include work inside factories that involves assembling-to-order (ATO) and work in many settings that involves engineering-to-order (ETO). This is in addition to work that is characterized by diverse settings, composition variety, and much uncertainty in agriculture and construction. Future research into realization of PLPA should be focused on such work where the productivity and consistency of outputs from human workers is currently low, but where not all work can be carried out by robots. Rather, there is need for development of hybrid intelligence systems involving human, cyborg and robot workers. This paper provides examples of how modelling situated entropy can inform development. In particular, hybrid intelligence systems that take into account the relative situated entropy of each type of worker: both before and after engineering efforts to reduce situated entropy.

## Figures and Tables

**Table 1:** Autonomous psychomotor work skills of human, cyborg and robot workers.

Worker Type	Autonomous Psychomotor Work Skills	
	Internal Action and External Action	Improvement Challenges
Human	Internal: Little, if any, conscious thought required in familiar settings. Internal: Little, if any, conscious through required in new settings. External: No supervision required External: Irreducible simplicity of fluid elegance in motions	Human autonomous psychomotor skills are in short supply. Requires instruction with demonstration followed by practice with feedback to enable autonomous psychomotor work skills. Yet, there are shortages of trainers who can provide demonstration and feedback.
Cyborg	Internal: More conscious thought required in familiar settings. Internal: More conscious through required in new settings. External: No supervision required External: More complexity and less elegance in motions	Enhancing technologies can immediately increase some human capabilities involved in psychomotor work skills, but they can increase the amount of conscious thought required.
Robot	Internal: Computational effort required in familiar settings. Internal: More computational effort required in new settings. External: Supervision required in new settings. External: Irreducible simplicity of fluid elegance in motions is possible.	Soft robotics, morphological computation, and learning by demonstration are not equal to the human capacity for autonomous psychomotor work skills enabled by least action internally and least action externally.

**Table 2:** Examples of realization of PLPA by human, cyborg and robot workers.

Worker Type	Work Example	Worker Type Advantage
Human	Agricultural work on slippery undulating sloping ground	*S_i_* is lowest by human reference to appropriate *w* for human general psychomotor abilities and *S_e_* is lowest because only human body weight is being maneuvered and it is maneuvered using human general psychomotor abilities.
Cyborg	One-of-a-kind construction work	*S_i_* is lowest by reference to appropriate *I* through AR, if it does not introduce higher than human *c_H_*(*t*), and *S_e_* is lowest because physical motion is supported by exoskeletons, if they do not introduce higher than human *c_I_*(*t*)
Robot	Soft product manufacturing work	Robotic innovations that minimize both *S_i_* and *S_e_*, rather than reducing one but increasing the other, can introduce lower *S* than very labor-intensive human practices that are not well-suited to improvement with cyborg technologies.
